# Acute insulin resistance in ST-segment elevation myocardial infarction in non-diabetic patients is associated with incomplete myocardial reperfusion and impaired coronary microcirculatory function

**DOI:** 10.1186/1475-2840-13-73

**Published:** 2014-04-05

**Authors:** Danijela Trifunovic, Sanja Stankovic, Dragana Sobic-Saranovic, Jelena Marinkovic, Marija Petrovic, Dejan Orlic, Branko Beleslin, Marko Banovic, Bosiljka Vujisic-Tesic, Milan Petrovic, Ivana Nedeljkovic, Jelena Stepanovic, Ana Djordjevic-Dikic, Milorad Tesic, Nina Djukanovic, Olga Petrovic, Olga Vasovic, Emilija Nestorovic, Jelena Kostic, Arsen Ristic, Miodrag Ostojic

**Affiliations:** 1 Department of Cardiology, Clinical Centre of Serbia, Ul. Koste Todorovica 8, 11000 Belgrade, Serbia; 2 School of Medicine, University of Belgrade, Belgrade, Serbia; 3 Center for Medical Biochemistry, Clinical Centre of Serbia, School of Pharmacy, University of Belgrade, Belgrade, Serbia; 4 Department of Nuclear medicine, Clinical Centre of Serbia, Belgrade, Serbia; 5 Institute of Medical Statistics and Informatics, School of Medicine, University of Belgrade, Belgrade, Serbia; 6 Institute for Gerontology and Palliative Care, Belgrade, Serbia; 7 Serbian Academy of Sciences and Arts, University of Belgrade, Belgrade, Serbia

**Keywords:** Insulin resistance, Acute myocardial infarction, ST-E resolution, Coronary microcirculation, Infarct size

## Abstract

**Background:**

Insulin resistance (IR) assessed by the Homeostatic Model Assessment (HOMA) index in the acute phase of myocardial infarction in non-diabetic patients was recently established as an independent predictor of intrahospital mortality. In this study we postulated that acute IR is a dynamic phenomenon associated with the development of myocardial and microvascular injury and larger final infarct size in patients with ST-segment elevation myocardial infarction (STEMI) treated by primary percutaneous coronary intervention (pPCI).

**Methods:**

In 104 consecutive patients with the first anterior STEMI without diabetes, the HOMA index was determined on the 2^nd^ and 7^th^ day after pPCI. Worst-lead residual ST-segment elevation (ST-E) on postprocedural ECG, coronary flow reserve (CFR) determined by transthoracic Doppler echocardiography on the 2^nd^ day after pPCI and fixed perfusion defect on single-photon emission computed tomography myocardial perfusion imaging (SPECT-MPI) determined six weeks after pPCI were analyzed according to HOMA indices.

**Results:**

IR was present in 55 % and 58 % of patients on day 2 and day 7, respectively. Incomplete post-procedural ST-E resolution was more frequent in patients with IR compared to patients without IR, both on day 2 (p = 0.001) and day 7 (p < 0.001). The HOMA index on day 7 correlated with SPECT-MPI perfusion defect (r = 0.331), whereas both HOMA indices correlated well with CFR (r = -0.331 to -0.386) (p < 0.01 for all). In multivariable backward logistic regression analysis adjusted for significant univariate predictors and potential confounding variables, IR on day 2 was an independent predictor of residual ST-E ≥ 2 mm (OR 11.70, 95% CI 2.46-55.51, p = 0.002) and CFR < 2 (OR = 5.98, 95% CI 1.88-19.03, p = 0.002), whereas IR on day 7 was an independent predictor of SPECT-MPI perfusion defect > 20% (OR 11.37, 95% CI 1.34-96.21, p = 0.026).

**Conclusion:**

IR assessed by the HOMA index during the acute phase of the first anterior STEMI in patients without diabetes treated by pPCI is independently associated with poorer myocardial reperfusion, impaired coronary microcirculatory function and potentially with larger final infarct size.

## Background

Full myocardial reperfusion with restoration of coronary microcirculatory function (CMF) is a therapeutic goal in ST-segment elevation myocardial infarction (STEMI) [1]. Despite successful primary percutaneous intervention (pPCI) it is not achieved in 30% to 50% of patients [2,3]. The reasons are complex, and include also several metabolic issues [3].

Insulin resistance (IR) as a part of metabolic syndrome is an important risk factor for the development of cardiac and vascular impairments [4,5] and carries ominous prognosis in the setting of acute myocardial infarction [6]. As a part of metabolic syndrome IR is associated with myocardial and microvascular injury after STEMI in clinical [7,8] and experimental studies [9]. Data connecting metabolic syndrome to the final infarct size are less clear [10-12]. However, emerging evidences support direct proatherogenic effects of IR [13-15], as well as its direct adverse effects on myocardial contractility [16]. As phenomenon *per se*, independent of other components of metabolic syndrome, IR was related to ischemic myocardial injury after elective PCI [17].

Recently, IR in the early phase of acute coronary syndrome in non-diabetic patients, assessed by the homeostatic model assessment (HOMA) index, was established as an independent predictor of in-hospital mortality [18-21]. This "acute" IR is a part of the acute glycometabolic response to stress, can be transient and can occur even in patients without chronic glycometabolic derangements [22]. Data regarding the relationships between acute IR and CMF after pPCI are scarce. Acute IR comprises acute hyperglycemia and/or acute hyperinsulinemia [23]. Hyperglycemia in STEMI patients has direct acute negative cardiovascular effects contributing to incomplete myocardial reperfusion and CMF impairment [24-28]. The prognostic relevance of hyperinsulinemia in STEMI patients [29,30] and its relationship with coronary flow [31] are less well evaluated and acknowledged.

We postulate that IR can occur in the early post pPCI period as a dynamic phenomenon even in non-diabetic patients, and be related to the development of microvascular and myocardial injury and final infarct size, independently of other variables. Accordingly, we have evaluated time dependent changes of IR in relation to myocardial reperfusion, CMF and final infarct size in non-diabetic STEMI patients treated by pPCI. We have defined coronary flow reserve (CFR), a marker of CMF, as a primary end-point. The residual ST-segment elevation (ST-E) and final infarct size were secondary endpoints.

The HOMA index is a simple and inexpensive marker of IR primary used in chronic states. It was recently validated against euglycemic hyperinsulinemic clamp in STEMI patients as feasible for assessing IR during myocardial infarction [32] and therefore used in the current study.

## Methods

### Patient population

The inclusion criteria for this prospective, single center, observational study included recanalization of left anterior descending artery (LAD) with visually assessed residual stenosis < 30%. The STEMI was defined and treated according to ESC guidelines [1]. Diabetes mellitus was defined according to the American Diabetes Association criteria [33] and patients without previously known diabetes but with HbA1c >6.5% on admission were not included. The exclusion criteria also included: (1) inability or refusal to obtain written informed consent; (2) inability to perform echocardiography examination with coronary flow reserve (CFR) measurements with adenosine due to hemodynamic instability, technically poor acoustic window precluding satisfactory imaging of the left ventricle (LV) or LAD flow by Doppler, or states known as contraindications for adenosine administration (chronic obstructive pulmonary disease, 2^nd^ or 3^rd^ AV blocks); (3) significant valvular disease, (4) temporary or permanent pacemaker and left bundle branch block precluding ECG analysis, (5) acute or chronic infection, (6) a history of autoimmune, malignant, liver, kidney or thyroid diseases. The Ethical committee of our hospital approved the study protocol. The patients were asked to participate in the study and to sign informed consent on admission to the hospital, before pPCI. Anthropometric parameters including weight and height were measured for all patients, and their body mass index (BMI) was calculated as ratio of weight to height squared. Hypertension was defined as systolic blood pressure ≥ 140 mmHg, a diastolic blood pressure ≥ 90 mmHg, or use of antihypertensive treatment. Patients were considered smokers if they smoked ≥ 1 cigarette/day at the time of admission or in the preceding 12 months. The Killip class, depending on the clinical manifestation of cardiac failure, was assessed on admission (Killip 1, no heart failure; Killip 2, S3 and/or basal lung crepitations; Killip 3, acute pulmonary edema; Killip 4, cardiac shock).

### Blood sampling and biochemical analysis

When a diagnosis of STEMI was established and a decision to perform pPCI was made, blood samples were obtained from a peripheral vein before starting intervention and after admission to the intensive coronary care unit every 6 h during the first 48 h and every 12 h during the rest of the stay in the intensive coronary care unit. Blood samples for troponin I (TnI), MB fraction of creatine kinase (CK-MB), N-terminal pro-brain natriuretic peptide (NT-proBNP), and high-sensitive C-reactive protein (hs-CRP) determination were allowed to coagulate at room temperature for 30 min, and then centrifuged at 4000 rpm for 10 min to obtain serum samples. Serum TnI and CK-MB were measured by chemiluminescent microparticle immunoassay (CMIA) on Architect i2000 System. The serum levels of NT-proBNP were measured by a Roche Cobas 6000 automated analyzer (Roche Diagnostics, Mannheim, Germany). The serum hs-CRP concentrations were measured by means of the immunoturbidimetric test (Olympus Life and Material Science Europe GmbH, Ireland) on an Olympus AU 400 analyzer. The lowest detectable level was 0.02 mg/L. Serum insulin was measured using a chemiluminescent microparticle immunoassay on an Architect i2000SR instrument (Abbott Diagnostics, Wiesbaden, Germany). According to the manufacturer’s package insert, Architect Insulin assay has a measurement range of 1–300 μU/mL with analytical sensitivity ≤ 1.0 μU/mL. Other biochemical and hematologic measurements involved the use of standard assays. The peak values for CK-MB, Tn I, NT-proBNP and hs-CRP were considered for further analysis and for other biochemical parameters 2^nd^ day values were used for analysis, because CFR was measured on the 2^nd^ day. The HOMA was calculated on the 2^nd^ and 7^th^ day according to the following formula: (fasting insulin [µU/mL] × fasting glucose [mmol/L])/22.5. Patients whose values exceeded the sex-specific 75th percentile (i.e., 1.80 for women and 2.12 for men) were considered to have HOMA-IR according to guidelines proposed by the European Group for the study of Insulin Resistance (EGIR) [34]. The estimated glomerular filtration rate (eGFR) was calculated using abbreviated Modification of Diet in Renal Disease study formula [35].

### Primary PCI

PCI was performed within 12 h from the onset of chest pain by experienced interventionists. Coronary angiography was performed as quickly as possible. Collateral flow from the patent vessels to the infarct-related artery was graded using the classification developed by Rentrop [36]. In all patients, a stent was successfully implanted in the LAD. Angiographic Thrombolysis In Myocardial Infarction (TIMI) flow grade was evaluated as described previously [37]. Slow/no-reflow was defined as TIMI ≤ 2 after PCI. All patients and their angiograms were graded according to the number of diseased coronary arteries. A coronary artery was considered diseased if there was any obstructive lesion ≥ 70% in diameter in that artery or one of its major branches (diameter ≥2.5 mm). Medication used in the acute phase included loading a dose of clopidogrel 600 mg and aspirin 300 mg and intravenous bolus of heparin before pPCI, followed by dual antiplatelet therapy (clopidogrel or ticlopidine and aspirin) and statins in all patients. ACE-I/ARB, beta blockers, diuretics, digitalis and calcium antagonist were used in 82%, 96%, 52%, 9%, 9%, respectively. Glicoprotein IIb/IIIa inhibitor, thrombectomy and intracoronary vasodilatators were administered at the discretion of the interventional cardiologist. In the current study 24% of patients had thiazide diuretics in therapy and 98% of patients had statins from the 3^rd^ day after pPCI. None of the patients included in the current study reached the glycaemic threshold of 11 mmol/l to get insulin.

### Outcome variables

CMF estimated by coronary flow reserve (CFR) was defined as a primary endpoint. Myocardial perfusion, assessed by the residual ST-E, and infarct size, evaluated by the fixed perfusion defect on SPECT-MPI, were defined as secondary endpoints.

### Electrocardiogram (*ECG)* measurements

The degree of myocardial reperfusion in this study was assessed by ECG ST-segment recovery, because it has strong power for predicting death, heart failure or shock up to 90 days after STEMI [37]. A standard 12-lead ECG was recorded at baseline and ≈ 30 minutes after pPCI. All ECG records were magnified and ST-E was measured at the J point to the nearest 0.05 mV. The person performed ECG measurement was blinded to other clinical and laboratory data. In the current study, we used a simple ST-segment- recovery method of residual ST-E measured in the most affected lead on the post-PCI ECG, since this method was previously shown to perform as well as complex ones in predicting outcomes after primary PCI in STEMI [38].

### Transthoracic echocardiographic examination with the CFR evaluation

CMF in this study was assessed by transthoracic echocardiography and CFR of the infarct, i.e. the left anterior descending coronary artery (LAD), since this method is noninvasive, reliable and validated for CMF assessment in post pPCI patients [39-41]. It was performed on the 2^nd^ day after pPCI using a commercially available ultrasound machine (Acuson Sequa C 256, Siemens, Medical Solutions, USA) and a 3V2C multifrequency transducer using second harmonic technology. Echocardiography included conventional resting 2D examination with measurements of LV end-diastolic and end-systolic volumes (LV EDV and LV ESV) and ejection fraction (EF) by Simpson’s methods, and evaluation of LAD CFR as previously described [39-41]. In short, color Doppler detection of the LAD flow was obtained by a modified apical approach using a 4-MHz transducer, with Doppler mapping velocity range set between 16 to 24 cm/sec. The systolic and diastolic coronary flow velocity spectrum was obtained at baseline and during the peak of hyperemia induced by i.v. adenosine (0.14 mg/kg/min during 1 minute). CFR was calculated as the ratio of hyperemic to basal peak diastolic flow velocities. All studies were digitally recorded and stored for off-line analysis by two experienced examiners blinded to patient clinical data. The intraobserver and interobserver variability for CFR LAD measurements in our laboratory is <10% [40].

### SPECT-MPI

Gated single-photon emission computed tomography myocardial perfusion imaging (SPECT-MPI) with 99 m-technetium metoxy-isobutile-isonitrile (99mTc-MIBI) was done 6 weeks after pPCI to define the final infarct size (percentage of myocardium with fixed perfusion abnormalities) by the person blinded to other clinical and laboratory data. 740 MBq of 99mTc-MIBI was injected 10–15 minutes after sublingual administration of 0.5 mg nitroglycerin coinciding with the peak hemodynamic response. The acquisition was performed 45–60 minutes after the injection. Gated SPECT-MPI data were acquired in the supine position with a single head SPECT gamma camera (Siemens, e.cam) equipped with a high-resolution low energy collimator. Sixty-four projection images were recorded over a 180° semicircular orbit extending from the 45° right anterior oblique position to the 45° left posterior oblique position, with matrix size 64 × 64, zoom 1.45, and gating 8 frames per cardiac cycle. Using the e.soft commercial software, transaxial tomograms were generated from gated projection data, reconstructed with a filtered back-projected algorithm, and reoriented to obtain oblique-angle tomograms parallel to the long and short axes of the left ventricle. The reconstructed data were projected as myocardial tomographic slices in short-axis, vertical-long, and horizontal-long axis views. The 4D-MSPECT software was then used for semiquantitative evaluation of myocardial perfusion and function. The extent of myocardial perfusion abnormalities (%) was expressed relative to the left ventricle, based on polar maps. A large infarction was defined as SPECT-MPI perfusion abnormality >20%.

### Statistical analysis

Continuous variables were tested for normal distribution using the Kolmogorov-Smirnov test. Normally distributed continuous variables are expressed with mean ± SD, and continuous variables that did not show a normal distribution are expressed as the median value and interquartile range (IQR: 25^th^, 75^th^ percentile). CFR was analyzed both as continuous variable (with normal distribution) and as categorical, with values ≥ 2 considered as normal. Worst residual ST-E was analyzed as categorical (<1 mm, 1 to <2 mm, and ≥ 2 mm). SPECT-MPI perfusion defect was analyzed as a continuous variable (without normal distribution) and categorical (>20% and ≤20%). Categorical data are shown as frequencies and percentages. For comparison between HOMA the positive vs. negative group student t-test, the χ^2^ test and the independent-samples Mann–Whitney U test were used. To account for multiple testing, Bonferroni’s correction was applied. Spearman’s rank correlation coefficients were used to assess the relationships between HOMA and other variables. Univariate and multivariate (backward Wald) binary logistic regression analysis were used to evaluate the relationship between outcome variables (residual ST-E ≥2 mm, CFR <2 and SPECT-MPI perfusion defect >20%) and potential determinants including the HOMA index. Results are expressed as the odd ratios (ORs) and their 95% confidence intervals (CI) per one standard deviation increment of each measure (facilitating comparisons of effect sizes for individual measures). The statistical analyses were performed using SPSS (SPSS version 20.0 Inc., Chicago). Statistical significance was defined as p < 0.05.

## Results

A total of 108 non-diabetic consecutive patients with first anterior STEMI treated by pPCI were initially screened for the study during the period from November 2009 to June 2010. Out of 108 initially screened patients 3 patients died before they completed the study and 1 was excluded due to poor acoustic window. The final study population included 104 patients. The SPECT-MPI studies were done in the subgroup of 61 patients who agreed to be examined and were able to come for this examination 6 weeks after pPCI. The incidence of HOMA index positivity was similar to that in the entire study group.

The median age in the study population was 57 years (IQR: 48–66) and 71% were male. They were mainly non smokers (67%), hypertensive (62%), had hypercholesterolemia (46%) and 65% had BMI >25 kg/m^2^. The median time from symptom onset to pPCI was 4 h.

Patient’s clinical and laboratory characteristics according to HOMA index positivity (i.e. IR) on day 2 and day 7 are listed in Tables 1, 2, 3 and 4.

**Table 1:** **Patients’ characteristics according to the 2**^**nd **^**day HOMA positivity**

		**2**^**nd **^**day HOMA index positive**	**2**^**nd **^**day HOMA index negative**	**p value**
	n, (%)	57/104 (55)	47/104 (45)	
Age	Median (IQR)	59 (50–68)	54 (48–64)	0.150
Male	n (%)	38 (66)	36 (77)	0.266
BMI (kg/m^2^)	Median (IQR)	25.2 (23.9-28.4)	25.9 (23.6-26.7)	0.809
Hypertension	n (%)	37 (65)	27 (57)	0.436
Hypercholesterolemia	n (%)	29 (51)	19 (40)	0.289
Smokers	n (%)	18 (32)	16 (34)	0.790
Symptom onset to pPCI (min)	Median (IQR)	257 (160–300)	210 (143–300)	0.156
Killip class on admission 1/2/3	n (%)	32/20/5 (56/35/9)	31/13/3 (66/28/6)	0.592
Heart rate (bpm)	Median (IQR)	81 (73–94)	74 (65–81)	0.010^a^
***Angiographic findings***				
Infarct related artery LAD	n (%)	57 (100)	47 (100)	ns
Multi vessel disease	n (%)	22 (39)	16 (34)	0.631
Initial (pre-stenting) TIMI flow 0/1/2/3	n (%)	46/8/1/2 (80/14/2/4)	38/2/5/2 (81/4/11/4)	0.106
Location of culprit lesion, proximal/mid/distal LAD	n (%)	24/33/0 (42/58/0)	27/20/0 (57/43/0)	0.119
Rentrop grade, 0/1/2-3	n (%)	34/10/3 (73/21/6)	50/7/0 (88/12/0)	0.059
***Procedural data***				
No of the implanted stents	Median (IQR)	1 (1–1)	1 (1–2)	0.154
Bare metal stent	n (%)	45 (79)	35 (75)	0.589
Final minimal stent diameter (mm)	Median (IQR)	2.9 (2.7-3.0)	2.8 (2.7-3.0)	0.910
*Post-procedural data*				
Slow/no reflow after pPCI	n (%)	9 (16)	3 (6)	0.135
***Echocardiography***				
LV EDV (ml)	Mean ± SD	112.5 ± 37.9	111.6 ± 36.1	0.903
LV ESV (ml)	Mean ± SD	58.9 ± 28.4	56.7 ± 25.5	0.684
LV EF (%)	Mean ± SD	49.8 ± 10.0	50.2 ± 10.5	0.846

IR, interquartile range; BMI, body mass index; pPCI, primary percutaneous coronary intervention; bpm, beat per minute; LAD, left anterior descending artery; TIMI, Thrombolysis in Myocardial Infarction; LV left ventricle; EDV, end diastolic volume; ESV, end systolic volume; EF, ejection fraction; SD, standard deviation.

^a^On the basis of Bonferroni’s correction, a p-value less than 0.025 indicates statistical significance.

**Table 2:** **Patients’ characteristics according to the 7**^**th **^**day HOMA positivity**

		**7**^**th **^**day HOMA index positive**	**7**^**th **^**day HOMA index negative**	**p**
	n (%)	60/104 (58)	44/104 (42)	
Age	Median (IQR)	60 (49–67)	51 (45–62)	0.117
Male	n (%)	42 (70)	32 (73)	0.762
BMI (kg/m^2^)	Median (IQR)	25.8 (25.0-28.7)	24.7 (22.1-26.6)	0.010^a^
Hypertension	n (%)	38 (63)	26 (59)	0.660
Hypercholesterolemia	n (%)	26 (43)	22 (50)	0.500
Smokers	n (%)	19 (32)	15 (34)	0.795
Symptom onset to pPCI (min)	Median (IQR)	250 (180–330)	185 (135–300)	0.031^a^
Killip class on admission 1/2/3/4	n (%)	32/23/5 (54/38/8)	31/10/3 (71/23/6	0.197
Heart rate (bpm)	Median (IQR)	80 (68–92)	78 (65–85)	0.010^a^
***Angiographic findings***				
Infarct related artery LAD	n (%)	60 (100)	44 (100)	ns
Multi vessel disease	n (%)	26 (43)	12 (27)	0.093
Initial (pre-stenting) TIMI flow 0/1/2/3	n (%)	50/4/2/4 (83/7/3/7)	34/6/4/0 (77/14/9/0)	0.122
Location of culprit lesion (proximal/mid/distal LAD)		29/31/0 (48/52/0)	22/22/0 (50/50/0)	0.867
Rentrop grade 0/1/2-3	n (%)	45/11/4 (75/19/6)	37/7/0 (85/15/0)	0.2193
***Procedural data***				
No of the implanted stents	Median (IQR)	1 (1–2)	1 (1–2)	0.863
Bare metal stent	n (%)	44 (73)	36 (82)	0.310
Final minimal stent diameter (mm)	Median (IQR)	2.9 (2.7-3.1)	2.8 (2.7-3.0)	0.744
***Post-procedural data***				
Slow/no reflow after pPCI	n (%)	10 (17)	2 (5)	0.056
***Echocardiography***				
LV EDV (ml)	Mean ± SD	117.2 ± 35.2	105.1 ± 38.4	0.100
LV ESV (ml)	Mean ± SD	63.5 ± 26.3	50.4 ± 26.4	0.014^a^
LV EF (%)	Mean ± SD	47.4 ± 8.4	53.4 ± 11.4	0.003^a^

IQR, interquartile range; BMI, body mass index; pPCI, primary percutaneous coronary intervention; bpm, beat per minute; LAD, left anterior descending artery; TIMI, Thrombolysis in Myocardial Infarction; LV left ventricle; EDV, end diastolic volume; ESV, end systolic volume; EF, ejection fraction; SD, standard deviation.

^a^On the basis of Bonferroni’s correction, a p-value less than 0.025 indicates statistical significance.

**Table 3:** **Patients’ laboratory data according to the 2**^**nd **^**day HOMA index positivity**

	**2**^**nd **^**day HOMA index positive (N = 57)**	**2**^**nd **^**day HOMA index negative (N = 47)**	**p**
Glucose (mmol/L)			
admission	7.8 (6.6-9.0)	7.6 (6.4-9.4)	0.667
2^nd^ day	5.7 (5.2-6.3)	4.9 (4.5-5.3)	<0.001^a^
7^th^ day	5.5 (4.9-5.9)	5.2 (4.9-5.6)	0.042
Insulin (IU/L)			
admission	18.82 (8.55-23.77)	11.29 (7.24-17.00)	0.009^a^
2^nd^ day	11.32 (8.88-13.52)	6.59 (4.78-7.50)	<0.00 1^a^
7^th^ day	10.81 (8.42-19.65)	8.80 (7.0-11.52)	<0.001^a^
HOMA index			
2^nd^ day	2.76 (2.21-3.42)	1.34 (1.04-1.69)	<0.001^a^
7^th^ day	2.75 (1.93-4.17)	2.04 (1.45-2.92)	<0.001^a^
HbA1c (%)	5.6 (5.4-5.8)	5.5 (5.3-5.7)	0.063
WBC count on admission (×10^9^/L)	12.7 (9.7-16.2)	12.6 (9.9-13.4)	0.739
hs-CRP (mg/L)	52.6 (19.9-123.7)	50.0 (22.3-108.8)	0.615
TnI (μg/L)	134.20 (48.38-165.00)	106.80 (79.84-190.17)	0.171
NT-proBNP (pg/mL)	2165 (1176–4693)	2099 (650–4133)	0.227
Total-C (mmol/L)	5. 22(4.40-6.03)	4.82 (4.53-5.68)	0.724
HDL-C (mmol/L)	1.08 (0.92-1.21)	1.16 (0.99-1.35)	0.825
LDL-C (mmol/L)	3.20 (2.65-3.80)	3.16 (2.91-3.69)	0.666
Apo A1 (g/L)	1.24 (1.11-1.36)	1.38 (1.15-1.53)	0.006^a^
Apo B (g/L)	1.00 (0.86-1.17)	0.98 (0.85-1.11)	0.418
TG (mmol/L)	1.60 (1.22-2.17)	1.45 (1.20-1.74)	0.138
Uric acid (μmol/L)	280 (231–332)	277 (209–329)	0.518
eGFR (mL/min per 1.73 m^2^)	89.06 ± 22.63	85.24 ± 23.05	0.398

Data are presented as median (interquartile range), except eGFR that is presented as mean ± standard deviation.

^a^On the basis of Bonferroni’s correction, a p-value less than 0.025 indicates statistical significance.

*Abbreviations:HOMA* Homeostatic Model Assessment, *HbA1c* hemoglobin A1c, *WBC* white blood cells, *hs-CRP* high sensitive C-reactive protein, *TnI* troponin I, *NTproBNP* N-terminal pro-brain natriuretic peptide, *Total-C* Total cholesterol, *HDL-C* high-density lipoprotein cholesterol, *LDL-C* low-density lipoprotein cholesterol, *Apo* apolipoprotein, *TG* triglycerides, *eGFR* estimated glomerular filtration rate, *SD* standard deviation.

**Table 4:** **Patients’ laboratory data according to the 7**^**th **^**day HOMA index positivity**

	**7**^**th **^**day HOMA index positive (N = 60)**	**7**^**th **^**day HOMA index negative (N = 44)**	**p**
Glucose (mmol/L)			
admission	7.9 (6.6-9.7)	7.6 (6.1-9.1)	0.134
2^nd^ day	5.6 (5.1-5.9)	4.9 (4.6-5.3)	<0.001^a^
7^th^ day	5.6 (5.2-5.9)	4.9 (4.8-5.3)	<0.001^a^
Insulin (IU/L)			
admission	12.37 (9.21-21.82)	10.53 (7.45-18.43)	0.056
2^nd^ day	8.70 (7.26-12.28)	7.56 (5.14-10.10)	0.017^a^
7^th^ day	14.52 (10.69-20.15)	7.28 (6.06-8.33)	<0.001^a^
HOMA index	5.5 (5.4-5.8)		
2^nd^ day	2.27 (1.76-2.98)	1.55 (1.09-2.28)	0.001^a^
7^th^ day	3.24 (2.54-4.19)	1.51 (1.21-1.83)	<0.001^a^
HbA1c (%)	5.5 (5.4-5.8)	5.6 (5.3-5.8)	0.801
WBC count on admission (×10^9^/L)	12.7 (10.0-16.2)	12.6 (9.7-13.2)	0.246
hs-CRP (mg/L)	57.3 (27.3-113.3)	38.1 (22.0-76.7)	0.030
TnI (μg/L)	135.86 (85.11-182.00)	78.36 (59.04-148.63)	0.019^a^
NT-proBNP (pg/mL)	2777 (1702–4693)	1157 (496–4150)	0.010^a^
Total-C (mmol/L)	4.24 (3.77-4.67)	4.26 (3.83-5.04)	0.613
HDL-C (mmol/L)	0.83 (0.70-0.99)	0.93 (0.82-1.12)	0.011^a^
LDL-C (mmol/L)	2.67 (2.39-3.09)	2.70 (2.21-3.47)	0.795
Apo A1 (g/L)	1.10 (0.96-1.30)	1.18 (1.10-1.25)	0.037^a^
Apo B (g/L)	0.85 (0.75-0.94)	0.90 (0.79-0.94)	0.481
TG (mmol/L)	1.39(1.11-1.85)	1.23 (0.97-1.64)	0.129
Uric acid (μmol/L)	273 (223–310)	279 (237–329)	0.703
eGFR (mL/min per 1.73 m^2^)	89.09 ± 25.23	84.95 ± 19.00	0.342

Data are presented as median (interquartile range), except eGFR that is presented as mean ± standard deviation.

^a^On the basis of Bonferroni’s correction, a p-value less than 0.025 indicates statistical significance.

*Abbreviations:HOMA* Homeostatic Model Assessment, *HbA1c* hemoglobin A1c, *WBC* white blood cells, *hs-CRP* high sensitive C-reactive protein, *TnI* troponin I, *NTproBNP* N-terminal pro-brain natriuretic peptide, *Total-C* Total cholesterol, *HDL-C* high-density lipoprotein cholesterol, *LDL-C* low-density lipoprotein cholesterol, *Apo* apolipoprotein, *TG* triglycerides, *eGFR* estimated glomerular filtration rate, *SD* standard deviation.

In the whole study group the HOMA index increased from day 2 (median 1.94; IQR: 1.42-2.85) to day 7 (median 2.33; IQR: 1.60-3.61) (p < 0.001), mainly due to the significant increment in insulin concentration. It increased from a median value of 8.37 IU/L (IQR: 6.63-11.65) on day 2 to a median value of 9.83 IU/L (IQR: 7.72-15.77) on day 7 (p < 0.001). Glycaemia was similar on day 2 (median 5.3; IQR 4.9-5.9 mmol/L) and day 7 (median 5.3; IQR: 4.9-5.8 mmol/L) (p = ns). According to EGIR (European Group for Insulin Resistance) criteria, 57 out of 104 patients (55%) had IR on the 2^nd^ day and 60 out of 104 patients (58%) had IR on the 7^th^ day.

### Relationship between insulin resistance and coronary microcirculatory function

CFR after pPCI was significantly lower in patients with IR based on the HOMA index both on the 2^nd^ (Table 5) and 7^th^ day (Table 6) (p = 0.002, for both). In HOMA index positive groups, both on day 2 and day 7 there were significantly more patients with impaired CMF (i.e. CFR < 2).

**Table 5:** **Outcome variables according to the 2**^**nd **^**day HOMA index positivity**

	**2**^**nd **^**day HOMA index positive**	**2**^**nd **^**day HOMA index negative**	**p**
***ECG*** *(n = 104)*	*(n = 57)*	*(n = 47)*	
Worst residual ST-E			0.001^a^
< 1 mm, n (%)	9 (16)	18 (38)	
1–2 mm, n (%)	6 (10)	11 (24)	
≥ 2 mm, n (%)	42 (74)	18 (38)	
***Echocardiography*** *(n = 104)*	*(n = 57)*	*(n = 47)*	
CFR, mean ± SD	1.71 ± 0.36	1.93 ± 0.37	0.002^a^
CFR < 2, n (%)	47 (83)	23 (49)	<0.001^a^
***SPECT-MPI*** *(n = 66)*	*(n = 36)*	*(n = 30)*	
Perfusion defect, %, (IQR)	38 (7–41)	29 (4–48)	0.957

HOMA, Homeostatic Model Assessment; ECG, electrocardiogram; ST-E, ST segment elevation; CFR, Coronary Flow Reserve; SD, standard deviation; SPECT-MPI, single-photon emission computed tomography myocardial perfusion image; IQR, interquartile range.

^a^On the basis of Bonferroni’s correction, a p-value less than 0.025 indicates statistical significance.

**Table 6:** **Outcome variables according to the 7**^**th **^**day HOMA index positivity**

	**7**^**th **^**day HOMA index positive**	**7**^**th **^**day HOMA index negative**	**p**
***ECG*** *(n = 104)*	*(n = 60)*	*(n = 44)*	
Worst residual ST-E			<0.001^a^
< 1 mm, n (%)	6 (10)	21 (48)	
1–2 mm, n (%)	13 (22)	3 (7)	
≥ 2 mm, n (%)	41 (68)	20 (45)	
***Echocardiography*** *(n = 104)*	*(n = 60)*	*(n = 44)*	
CFR, mean ± SD	1.71 ± 0.39	1.95 ± 10.33	0.002^a^
CFR < 2, n (%)	48 (80)	22 (50)	0.001^a^
***SPECT-MPI*** *(n = 66)*	*(n = 37)*	*(n = 29)*	
Perfusion defect, %, median (IQR)	39 (32–50)	20 (0–43)	0.027

HOMA, Homeostatic Model Assessment; ECG, electrocardiogram; ST-E, ST segment elevation; CFR, Coronary Flow Reserve; SD, standard deviation; SPECT-MPI, single-photon emission computed tomography myocardial perfusion image; IQR, interquartile range.

^a^On the basis of Bonferroni’s correction, a p-value less than 0.025 indicates statistical significance.

**Figure 1:**
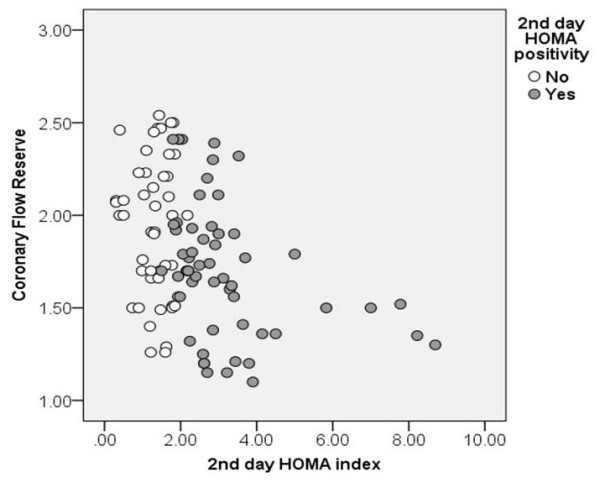
**Relationship between 2**^**nd **^**day HOMA index and coronary microcirculatory function, assessed as coronary flow reserve after pPCI.**

CFR significantly correlated with both HOMA indices (for 2^nd^ day HOMA: r = -0.386 and for 7^th^ HOMA: r = -0.331, p < 0.001) (Figure 1). CFR correlated also with 2^nd^ day glycaemia (r = -0.238, p = 0.015) and 2^nd^ day insulinemia (r = -0.297, p = 0.002). CFR did not correlate with HbA1c (r = 0.007, p = 0.941), weakly correlated with glycaemia on admission (r = -0.187, p = 0.054) and did not correlate with insulinemia on admission (r = 0.007, p = 0.946).

In multivariable model 2^nd^ day HOMA was an independent predictor of impaired CFR after adjustment for significant univariate predictors (heart rate, TnI peak, hsCRP), previously shown predictors of myocardial reperfusion (time from symptom onset to pPCI, glucose on admission, NT-proBNP) and potential cofounding variables for IR (age, gender and BMI), with OR of 5.98 (95% CI 1.88-19.03, p = 0.002).

### Relationship between insulin resistance and degree of myocardial reperfusion

The residual ST-E ≥ 2 mm on post-procedural ECG was significantly more frequent among patients with IR compared to those without IR, both on day 2 (Table 5) and day 7 (Table 6).

The 2^nd^ day HOMA index remained independently associated with residual ST-E ≥2 mm in multivariable analysis adjusted for significant univariate ST-E predictors (BMI and age), potential confounding variables for IR (gender) and previously shown predictors of myocardial reperfusion after primary PCI (time from symptom onset to pPCI, glycaemia on admission), with the OR of 11.70 (95% CI 2.46-55.51, p = 0.002).

### Relationship between insulin resistance and final infarct size

In 66 out of 104 patients SPECT-MPI was done to assess the final infarct size 6 weeks after pPCI. Patients with IR based on the 7^th^ day HOMA index positivity had a larger perfusion defect compared to patients without IR (p = 0.027), however the difference was of borderline statistical significance when Bonferroni’s correction was applied (Table 6). SPECT-MPI perfusion defect correlated with 7^th^ day HOMA index (r = 0.331, p = 0.008), but not with the 2^nd^ day HOMA index.

In the multivariable model the 7^th^ day HOMA index was an independent predictor of a large infarction (i.e., perfusion defect >20%) after adjustment for significant univariate predictors (TnI peak, time form symptom onset to pPCI), previously shown predictors of myocardial reperfusion (glucose level on admission) and potential cofounding variables for IR (age, gender and BMI) with OR of 11.37 (95% CI 1.34-96.21, p = 0.026).

### Correlations between HOMA indices with biochemical and clinical parameters

The HOMA index on day 2 correlated with the HOMA index on day 7 (r = 0.406, p < 0.001). The second day HOMA index significantly correlated with heart rate (r = 0.354, p < 0.001), peak values of hs-CRP (r = 0.205, p = 0.037), NT-proBNP (r = 0.230, p = 0.02) and Tn I (r = 0.258, p = 0.009). The seventh day HOMA index was significantly associated with heart rate (r = 0.205, p = 0.02), left ventricular ejection fraction (r = -0.241, p = 0.02), BMI (r = 0.231, p = 0.019) and peak values of hs-CRP (r = 0.290, p = 0.003), NT-proBNP (r = 0.321, p = 0.001) and Tn I (r = 0.235, p = 0.017).

## Discussion

The present study analyzed IR in the acute phase of STEMI in non-diabetic patients treated by pPCI. First, it confirms that IR in the early post pPCI period is common even in non-diabetic patients and changeable. Second, IR was related to the degree of myocardial reperfusion and correlated well with the CMF after mechanical revascularization, independently of other variables. Third, IR at the end of the first week after pPCI was an independent predictor of the final infarct size.

IR in the acute phase of STEMI is predominately part of acute glycometabolic response to stress [22]. The term *acute* IR implies its presence during acute (early) phase of STEMI. Although we included only patients without diabetes mellitus, we cannot strictly exclude the presence of IR before and/or after the first STEMI week, since evaluation of IR status was not done before or after this period. Generally, in critically ill patients acute IR is related to more severe acute illness and warrants poor clinical outcome [42,43]. By traditional concept, acute IR is induced by acute stress, with a small possibility to be further directly involved in the development or aggravation of acute illness. However, recent studies [18-21] challenge this concept to a certain extent.

Few studies have analyzed time dependent changes of acute IR and/or insulin sensitivity and have related it to clinical outcomes [22,43]. Nishio et al. [22] performed serial HOMA index measurements among patients going to pPCI and identified those with transient IR, in whom HOMA index correlated with stress hormones and patients with persistent IR, in whom HOMA index during the 4 months follow-up correlated with leptin and contributed to stent restenosis. In the current study acute IR was present in 55% of patients on the 2^nd^ day and in 58% of patients on the 7^th^ day. The HOMA index slightly, but statistically significantly increased from day 2 to day 7, mainly due to the increment in insulin concentration. Similar increment in insulinemia in the early post PCI period in non-diabetic patients was previously reported [44]. The potential mechanisms are not clear. Effects of prolonged cardiovascular (hemodynamic) stress and inflammation induced by STEMI could be the reasons [44]. In our study the 7^th^ day HOMA index correlated better than the 2^nd^ day HOMA index with the peak values of NT-proBNP and hs-CRP. Nutritional status might also influence the IR increment. In our study the majority of patients were on regular nutrition on the 7^th^ day as opposed to the 2^nd^ day, with a higher fat intake and potentially higher free fatty acids effects on the insulin sensitivity [45,46]. However, a comprehensive nutrition data analysis was not done. The effect of drugs should not be neglected, including those of thiazide diuretics, beta blockers and some statins [47-49], reported to have potential impact on insulin sensitivity. However, in the current study we could not find significant differences between HOMA positive and HOMA negative patients regarding the drug regime. None of the patients reached the glycaemic threshold of 11 mmol/l to get insulin.

One of the main findings in the current study is that acute IR was related to more impaired CMF after pPCI, independently of other potential covariates. CFR is a sensitive parameter of CMF. CMF after STEMI is important for the clinical outcome independently of epicardial flow [50]. Although the inverse relationship between HOMA index and CFR was described in different states [51-53], to the best of our knowledge the current study is the first to link acute IR assessed by the HOMA index and CFR in non-diabetic STEMI patients. Acute hyperglycemia *per se* predicted impaired epicardial flow before pPCI [54] and impaired CMF after pPCI in unselected, mixed diabetic and non-diabetic patients [55]. It is related to increased oxidative stress in STEMI, increases vascular cell apoptosis and induces acute endothelial dysfunction [56,24,57]. Insulin is under healthy condition coronary vasodilatator and in the dose-dependent manner increases myocardial blood flow [58,59]. However, these effects are abolished in chronic, obesity related IR [60,61]. Only one study evaluated acute insulinemia and coronary flow in acute coronary syndrome. In 60 patients admitted to the emergency department for suspected acute coronary syndrome acute hyperinsulinemia was present in half of them, and closely correlated with TIMI coronary flow score [31]. In the current study CFR better correlated to the HOMA index than to acute glycaemia and/or insulinemia and in the multivariable model including admission glycaemia, the HOMA index remained an independent CFR predictor. These data might suggest that acute IR as a phenomenon *per se* could be related to the development of CMF impairment after pPCI.

The relationship between acute IR and ST-E resolution was not previously evaluated. Acute hyperglycemia was related to limited ST-E recovery in mixed diabetic and non-diabetic STEMI population after thrombolytic therapy [62] and after pPCI [63]. Metabolic syndrome (chronic IR) was a predictor of ST-E resolution after pPCI [11]. In the current study in patients without diabetes, incomplete ST-E resolution was significantly more frequent among those with acute IR. However, only the 2^nd^ day HOMA index was independently associated with the residual ST-E.

The relationship between acute IR in non-diabetic STEMI patients and myocardial damage in terms of peak enzymes was previously reported [18,19]. In our study both HOMA indices correlated well with the peak TnI. However, only the 7^th^ day HOMA index was an independent predictor of the final infarct size on SPECT-MPI. It could be speculated that prolonged IR during acute phase is important. Furthermore, the 7^th^ day HOMA index might not only be a part of acute glycolmetabolic response to stress, but also part of chronic or prolonged intermittent IR that might influence formation of final infarct size in the following weeks after STEMI.

From the clinical standpoint, in the current study HOMA index on day 2 better correlated with myocardial reperfusion, both HOMA indices well correlated with the CMF, whereas HOMA index on day 7 predicted final infarct size. Both data might be clinically valuable: HOMA index on day 2 for the in-hospital course, HOMA index on day 7 for the long-term processes such as formation of the final infarct size. Although our data do not unambiguously demonstrate cause and effect relationship between acute IR and CMF, they strengthen the need for attention to be paid to IR during and after STEMI. Taking in mind that IR could be potentially modified, its regulation might become a therapeutic target both in acute STEMI and afterwards.

### Study limitations

Study results should be interpreted with some limitations. First, our study is a single centre experience with a relatively small sample size. Second, CMF was measured by echocardiography without invasive or magnetic resonance imaging. However, this approach has been previously validated in our laboratory or elsewhere [39-41] and compared to positron emission tomography imaging [39]. Third, IR assessment was based on the HOMA index and EGIR classification, which was primarily defined for chronic states. Whether different cut-offs for acute IR should be used in the acute phase of myocardial infarction, as suggested by some authors [21], remains an open question. In the current study the "area at risk" was not determined by SPECT-MPI. However, there were no significant differences between patients with IR vs. without IR regarding the infarct related artery, the position of the culprit lesion and collateral circulation.

## Conclusion

In conclusion, our results suggest that acute IR after pPCI in non-diabetic STEMI patients is related to impaired myocardial reperfusion, impaired CMF and potentially to a larger final infarct size.

## Abbreviations

IR: Insulin resistance; STEMI: ST segment elevation myocardial infarction; HOMA: Homeostatic model assessment; pPCI: Primary percutaneous coronary intervention; CMF: Coronary microcirculatory function; LAD: Left anterior descending artery; HbA1c: Hemoglobin A1c; CFR: Coronary flow reserve; LV: Left ventricle; TnI: Troponin I; NT-proBNP: N-terminal pro-brain natriuretic peptide; hs-CRP: High-sensitive C-reactive protein; eGFR: Estimated glomerular filtration rate; TIMI: Thrombolysis in myocardial infarction; ST-E: ST segment elevation; SPECT-MPI: Single-photon emission computed tomography myocardial perfusion image; LV EDV: Left ventricular end diastolic volume; LV ESV: Left ventricular end systolic volume; LV EF: Left ventricular ejection fraction; IQR: Interquartile range; OR: Odd ratio; CI: Confidence interval; WBC: White blood cells; HDL: High-density lipoprotein; LDL: Low-density lipoprotein; Apo: Apolipoprotein.

## Competing interests

The authors declare that they have no competing interest.

## Authors’ contributions

DT: conceived the study, participated in the design of the study, performed echocardiographic and CFR examinations, helped to perform statistic analysis and helped to write the manuscript. SS: carried out laboratory analysis and helped to write the manuscript. DSS: carried out SPECT-MPI analysis and helped to write the manuscript. MP: helped in ECG analysis. BB carried out cath lab work, angiographic analysis, and reviewed the manuscript. MB helped in echocardiographic examinations and cat lab work. JM participated in the design of the study, performed statistical analysis. DO performed cath lab work and angiographic analysis. BVT supervised echocardiographic examinations. MP performed echocardiographic examination. IN: performed echocardiographic examination. JS: participated in the design of the study. ADD: helped in CFR measurements. MT: helped in CFR measurements. ND: helped to carry out laboratory analysis. OP: performed echocardiographic examination and CFR measurements. OV: helped in ECG analysis. EN: helped in echocardiographic examination. JK: carried out cath lab work and angiographic analysis. AR: helped in coordination. MO: participated in the design of the study. All authors read and approved the final manuscript.
